# High-Resolution Vessel Wall Images and Neuropsychiatric Lupus: A Scoping Review

**DOI:** 10.3390/diagnostics15070824

**Published:** 2025-03-25

**Authors:** Bruno L. D. Matos, Luiz F. M. Borella, Fernanda Veloso Pereira, Danilo Rodrigues Pereira, Simone Appenzeller, Fabiano Reis

**Affiliations:** 1 Department of Radiology and Oncology, School of Medical Sciences, Universidade Estadual de Campinas (UNICAMP), Campinas 13083-970, Brazil; bruno112358@gmail.com (B.L.D.M.); borella.luiz@gmail.com (L.F.M.B.); fernandavelosop@gmail.com (F.V.P.); fabianoreis2@gmail.com (F.R.); 2 Autoimmunity Lab, School of Medical Sciences, Universidade Estadual de Campinas (UNICAMP), Campinas 13083-970, Brazil; danilorp@unicamp.br; 3 Department of Orthopedics, Rheumatology and Traumatology, School of Medical Sciences, Universidade Estadual de Campinas (UNICAMP), Campinas 13083-970, Brazil

**Keywords:** high-resolution vessel wall imaging, intracranial vessel wall, central lupus, lupus arteritis, lupus vasculitis

## Abstract

**Background**: Systemic lupus erythematosus (SLE) is a multisystem autoimmune disorder. Neuropsychiatric manifestations are frequently observed and are associated with increased morbidity and reduced quality of life. Magnetic resonance imaging (MRI) is the neuroimaging procedure of choice for investigation. High-resolution vessel wall imaging (HRVWI) is a neuroimaging methodology that allows active mapping of pathophysiological processes involving brain vessel walls. **Methods**: To exemplify the importance of HRVWI and its usefulness in patients with SLE, we carried out a scoping review (following PRISMA guidelines) using the PubMed and Embase databases. **Results**: We retrieved 10 studies that utilized HRVWI in neuropsychiatric SLE, including a total of 69 patients. The majority, 84% (58/69), were women, with ages ranging between 16 and 80 years (average 38.4 years). Approximately 46.3% (32/69) of patients had white matter lesions in the brain at the time of investigation, and 77% (53/69) had normal magnetic resonance angiography. Treatment with immunosuppressants led to the resolution of the majority of the findings. **Conclusions**: Imaging plays an important role in investigating neuropsychiatric SLE. HRVWI analysis is gaining more importance, with its ability to identify inflammation even if angiographic MRI sequences (3D TOF) are normal, allowing the institution of early immunosuppressant treatment and resolution of symptoms.

## 1. Introduction

Systemic lupus erythematosus (SLE) is a multisystemic autoimmune disease that significantly impacts quality of life. Neuropsychiatric lupus occurs in up to 75% of patients during the disease and represents a heterogeneous range of manifestations with varying severity that reflects distinctive pathophysiological mechanisms [1,2,3].

Vasculitis of the central nervous system (CNS) is a rare SLE manifestation. It occurs secondary to complement activation and immune complexes deposition. This leads to the activation of endothelial cells that promote the recruitment of monocytes and the induction of pro-inflammatory cytokines, with consequent inflammation of the vessel wall. The presence of segmental inflammation can induce platelet activation and, additionally, thromboembolic complications [4].

In general, neuropsychiatric manifestations in SLE are divided into inflammatory and/or ischemic in origin, and magnetic resonance imaging (MRI) is one of the most recognized methods for investigation [5]. When present, vasculitis frequently affects the small vessels of the CNS, and magnetic resonance angiography (MRA) is often normal. Typical findings on MRI are hyperintense white matter (WM) lesions, which represents the collective term referring to small or confluent T2-FLAIR hyperintensities, including the WM, basal ganglia, cerebellum, and brainstem [1]. Involvement of large and medium vessels is less frequent [6,7]. Cerebrospinal fluid (CSF) can be normal or show increased protein levels in neuropsychiatric SLE [8].

The pathology of vasculitis in SLE patients has not been fully understood due to the scarcity of biopsy or postmortem brain specimens [6,7]. Some MRI studies have shown large cerebral vessel involvement in SLE patients associated with thrombus, dissection, fibromuscular dysplasia, or atherosclerosis.

The diagnosis of vasculitis remains challenging, especially in patients presenting with nonspecific symptoms or signs of diffuse CNS manifestations. There is a wide variety of symptoms of cerebral vasculitis, including neurological and/or psychiatric symptoms. Acute cognitive changes can be an important finding. MRI is the exam of choice due to its high tissue contrast. The use of different sequences can help to elucidate pathological changes. Luminal images are already known to aid in intracranial vasculitis diagnosis, but their sensitivity may be reduced when small vessels are affected [9,10,11].

This paper aims to review the use of high-resolution vessel wall imaging (HRVWI) in neuropsychiatric SLE to define its utility in clinical practice. In addition, we aim to determine the associated clinical and immunological features in SLE patients presenting HRVWI abnormalities and to determine the correspondence between the angiographic findings and vessel wall analysis. Since neuropsychiatric manifestations in SLE can be secondary to non-SLE-related conditions, we also present the HRVWI findings in neuropsychiatric mimics such as infections, aneurysm dissection, and reversible cerebral vasoconstriction syndrome (RVCS).

## 2. Materials and Methods

The search was performed according to the “Preferred Reporting Items for Systematic Reviews and Meta-Analyses” (PRISMA) guidelines for scoping reviews (PRISMA-ScR) [12].

A search of the PubMed and Embase databases, without data restrictions, was limited to articles in English for the terms “high-resolution vessel wall” and “vessel wall”, which were combined using the Boolean operator “AND” with the keyword “systemic lupus erythematosus”, and was performed on 3 August 2024. Two independent investigators reviewed all abstracts. The inclusion criteria for full-text review were (1) clinical diagnosis of SLE [13] or articles addressing neuropsychiatric SLE aspects and (2) patients evaluated with vessel wall sequences through MRI or studies that address aspects of this examination. The exclusion criteria covered publications that (1) did not contain important clinical information regarding disease activity or neuropsychiatric manifestations or (2) did not perform vessel wall analysis. Data were collected using a standardized review protocol by two investigators separately. A third investigator resolved conflicts. Variables collected included the number of patients, sex, age, SLE presenting features, neuropsychiatric symptoms, location of MRI abnormality, MRA and HRVWI findings, highlight patterns, diagnosis, treatment, and response.

**Figure 1:**
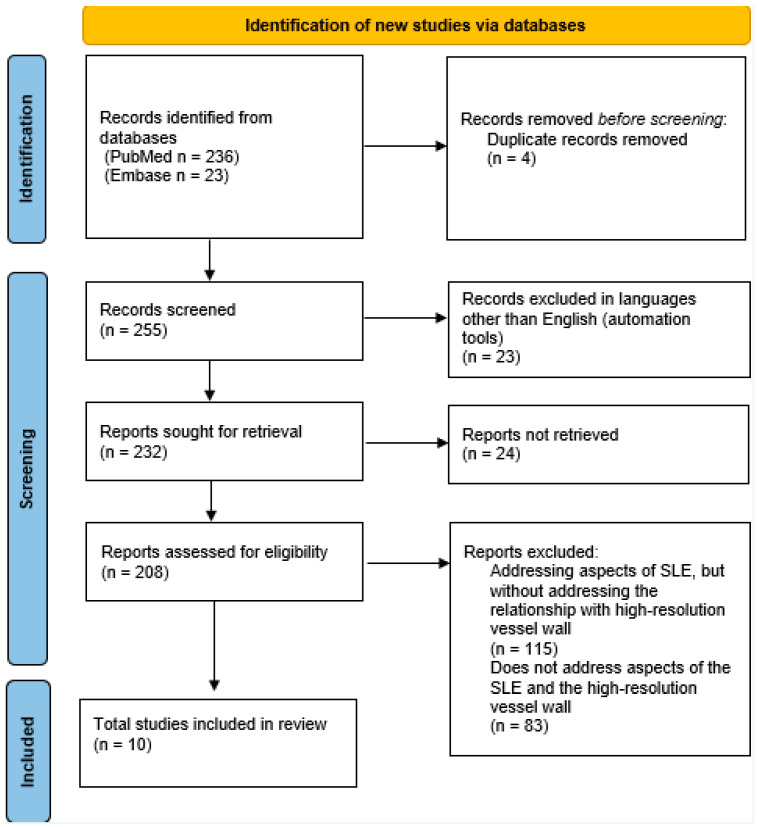
Search strategy.

This search protocol was not registered. The search process is illustrated in Figure 1.

## 3. Results

We found 10 studies demonstrating the association between HRVWI and neuropsychiatric SLE with a total of 69 patients (Table 1). The time between SLE diagnosis and symptoms averaged 10.5 years, with the minority of patients having neuropsychiatric symptoms and HRVWI at disease onset. Of the 69 patients, 58 (84%) were women, with ages between 16 and 80 years (average of 38.4 years). All patients had neuropsychiatric symptoms that varied, including headache, dizziness, blurred vision, auditory and visual hallucinations and gait change, mental confusion, aphasia, and hemiparalysis.

MRI was abnormal in 46.3% (32/69) of patients and included white matter hyperintensity, diffuse atrophy, and brain infarcts located in different segments of the brain. Infarcts were described in 19 of 32 (59%) patients and varied from lacunar, border, and localized cortical infarcts to large territorial infarcts.

All patients underwent imaging studies, including contrast-enhanced HRVWI. Regarding the enhancement pattern, of the studies that provided this information, approximately 6/9 (66.6%) patients showed concentric and multifocal enhancement, with one concentric and unifocal enhancement. One patient with encephalitis and one with reversible vasoconstriction syndrome (RCVS) did not demonstrate enhancement on HRVWI. In 1 of the studies that verified alterations in the analysis of the vessel wall in 60 patients, the enhancement pattern was demonstrated through the affected segments, in which the concentric pattern affected 554/571 segments (97%), while in 17 (3%), they had eccentric and irregular enhancements. Furthermore, 53 of 67 (79%) patients with abnormalities on HRVWI did not demonstrate abnormalities on time-of-flight (TOF) mMRA.

All patients were treated with corticosteroids and/or cyclophosphamide or other immunosuppressants. In four studies, antiplatelet/anticoagulant treatment was added to immunosuppressive therapy. The time between the start of therapy and clinical/radiological improvement was identified in five studies, with the time ranging from 1 to 12 weeks.

Short-term outcomes were reported in all studies. Follow-up was reported in only nine studies, with some improvement in the clinical picture and/or imaging findings (partial/total) in seven patients (77.7%).

## 4. Discussion

Neuropsychiatric manifestations have been reported in 10–80% of SLE patients, depending on the method used, and are associated with increased morbidity, mortality, and a reduced quality of life [23]. The impact on morbidity and quality of life varies according to the type of neurological manifestation, ranging from mild (e.g., headache) to severe, such as cerebrovascular disease and organic brain syndrome. This variation in severity depends on the extent of ischemic and/or autoimmune/inflammatory mechanisms. In cases of vasculitis, endothelial injury and complement activation can be observed [24].

The assessment of the vessel wall is gaining recognition for its ability to identify early changes, increase diagnostic accuracy, and potentially reflect inflammatory activity. The enhancement pattern of the vessel wall can differentiate between autoimmune and infectious diseases, making it an important adjunct to established diagnostic modalities (Table 2) [25]. The presence of concentric, homogeneous wall thickening and enhancement of intracranial arteries on HRVWI does not allow for differentiation between primary and secondary vasculitis (Table 2). Other conditions that may mimic vasculitis, such as reversible cerebral vasoconstriction syndrome (RCVS), sarcoidosis, and lymphoproliferative diseases, must be excluded [25]. In infectious vasculitis, HRVWI demonstrates various stenosis patterns and concentric or eccentric vessel wall thickening, typically with contrast enhancement [25].

An interesting finding was that only four patients had symptoms at disease onset. The majority of patients had more than 5 years of disease. Inflammatory features were typically described as occurring within the first two years of the disease [1,4]. There are various classification criteria for vasculitis, with those most commonly used being based on its cause or location. Primary vasculitis occurs in the absence of systemic features and is classified according to vessel size (large, medium, and small vessels) or as variable single-organ vasculitis [26,27]. The term “systemic vasculitis” is used when systemic features are present, as seen in SLE.

The blood–brain barrier, the meningeal barrier, and the blood–cerebrospinal fluid (CSF) barrier are the three primary neuroimmune interfaces that protect against toxins and have been increasingly associated with neuropsychiatric SLE in studies. Disruptions to these barriers are suggested by the pro-inflammatory characteristics of CSF, including elevated protein and immunoglobulin levels as well as increased cytokines such as interleukin-6 (IL-6) [28]. However, these changes are not specific and can also be observed in other inflammatory conditions [28,29,30]. Once inflammatory cytokines, B cells, and T cells enter the brain, they activate microglia or directly induce neuronal death. Autoantibodies, particularly anti-endothelial cell antibodies, anti-ribosomal P protein antibodies, and antiphospholipid antibodies, have also been implicated in neuropsychiatric SLE [28,29,30].

Endothelial damage in SLE is secondary to complement activation and the deposition of immune complexes in brain vessels, accompanied by monocyte infiltration into the arterial wall. This results in short segments of affected tissue interspersed with areas of normal vessel walls [28,29,30]. Identifying the etiologic mechanism in neuropsychiatric SLE is critical, as it is directly linked to targeted treatment strategies. MRI is the gold standard for evaluating SLE patients with neuropsychiatric manifestations. Current MRI protocols include distinct imaging sequences specifically designed for this purpose

In vasculitis and/or vasculopathy, MRI typically reveals hyperintense regions on T2/FLAIR-weighted images, which appear hypointense on T1-weighted images in the cerebral white matter. Lesions can also be present in the brainstem and basal ganglia [30]. These hyperintense and hypointense areas are often punctate and isolated, though they may become confluent, and they are typically non-enhancing. White matter lesions are observed in 30–75% of patients with neuropsychiatric SLE (NPSLE) [30]. These lesions can be associated with active clinical or subclinical neuropsychiatric involvement [1,30,31]. When infarcts occur in NPSLE patients, they often appear in multiple locations, suggesting a high recurrence rate of ischemic events. In such cases, diffusion-weighted imaging can help determine whether the infarcts are acute, subacute, or chronic, as restricted diffusion may be seen in acute lesions due to cytotoxic edema [1].

(1H) magnetic resonance (MR) spectroscopy allows for noninvasive in vivo quantification of metabolite concentrations in brain tissue. N-acetyl-aspartate (NAA) is predominantly found in higher concentrations in neurons and axons, making it a marker of neuronal viability and axonal integrity. NAA levels are reduced in chronic lesions in patients with systemic lupus erythematosus (SLE) and brain involvement. Additionally, active SLE is characterized by a reduction in NAA (and the NAA/creatine (Cr) ratio), which may be transient and reflect axonal dysfunction after disease control [1,32].

Creatine (Cr) is the most stable metabolite and serves as a marker for cellular metabolism via the Cr kinase reaction [1,32]. Choline (Cho) is a marker of cell membrane turnover (synthesis and degradation). Increased Cho levels are associated with inflammation and disease activity [1,32]. Changes in Cho levels have also been observed in normal-appearing brain parenchyma preceding neurological manifestations, suggesting that these metabolites may predict cerebral involvement in SLE [1,33]. Elevated Cho is seen in active myelin destruction and is linked to gliosis, vasculopathy, and edema, as demonstrated in the histopathology of neuropsychiatric SLE (NPSLE) [1,32,33].

As mentioned, VW-MR imaging often demonstrates smooth, homogeneous, concentric arterial wall thickening and enhancement in patients with central nervous system (CNS) vasculitis, differentiating it from atherosclerotic plaque. Ide et al. [3], evaluating 60 patients, identified various types of vessel-wall focal lesions, which were more frequent in the SLE group and more prominent in the initial segments of the middle cerebral artery.

Vessel wall analysis requires proper image acquisition techniques, the radiologist’s expertise in interpreting findings, and a reliable clinical history, as variations or artifacts in image acquisition may be misinterpreted as pathological changes. For example, several potential pitfalls can occur during this sequence: (1) Adequate blood suppression relies on appropriate flow velocity. When blood flow is low, such as in cases of aneurysms, blood hypoperfusion, or arterial dilation, areas of incomplete blood flow suppression may occur, potentially mimicking enhancement. (2) The vasa vasorum and adjacent veins can also be mistaken for vessel wall enhancement. (3) Thrombectomy, due to its mechanical effects, can induce thickening and concentric enhancement, which may resemble an abnormality consistent with vasculitis [34,35,36,37].

The ideal acquisition time is still the subject of study; currently, what is most described in the literature is between 5 and 10 min after contrast administration. Before 5 min, studies indicate a weak acquisition of enhancement, and evidence of acquisition 10 min after administration is limited. As the time to perform the vessel wall analysis sequence is generally between 5 and 7 min, 5 min between contrast and acquisition is appropriate [36].

In atherosclerosis, the wall abnormality is typically nonconcentric and heterogeneous [37]. Vasculitis may also result in eccentric wall abnormalities [37]. Additional vessel wall features of atherosclerotic plaque on T1-, T2-, and post-contrast images can help distinguish between vasculitis and plaque. It is thought that arterial wall enhancement in patients with CNS vasculitis reflects increased endothelial permeability, with contrast leakage from the lumen into the arterial wall. Vasa vasorum-related contrast leakage is a potential mechanism for this enhancement [37].

However, it is important to mention that in patients with lupus, the white matter lesions may be associated with vasculitis and the atherosclerosis associated with SLE pathology, as accelerated atherosclerosis occurs in SLE patients [3].

Vascular wall analysis is an emerging technique that shows significant promise in evaluating intracranial vasculopathy and is increasingly used to complement conventional angiographic imaging techniques. It enhances diagnostic specificity by detecting changes in the vascular wall, non-stenotic lesions, and lesions in distal vessels that are identified but not visible on luminal imaging [5].

The primary technical parameters for obtaining images for vessel wall analysis include the following: (1) A 3-Tesla field (or higher) for high resolution and better signal strength enables time-efficient, high-resolution acquisition due to its high spatial resolution. This is necessary because the thickness of the distal internal carotid artery is 0.2–0.4 mm, and the middle cerebral and basilar arteries range from 0.2 to 0.3 mm, with the lumen being 1/10 of the vessel diameter. Seven-Tesla MRI scanners have also been used to optimize protocols. (2) Acquisitions through 3D Fast Spin Echo T1 Black Blood Imaging sequences in both the short and long axis. (3) Multiple tissue weightings. (4) Signal suppression in luminal blood, cerebrospinal fluid (CSF), and adjacent brain parenchyma to enhance the demonstration of the vessel wall. The procedure is generally performed using 8-, 12-, or 16-channel head coils, though using 32 or 64 channels can improve the periphery of the field of view [37,38,39,40,41].

Before analyzing the changes that can be identified when analyzing the vessel wall, it is necessary to understand the normal pattern, as comparative analysis is of fundamental importance, especially in non-focal changes [38]. However, a healthy wall is thinner than a pathological wall and is more difficult to identify. Therefore, the resolution capacity needs to be high to allow visible limits between the walls and the internal and external interfaces [39]. The normal appearance should have regular contours, uniformly diffuse thickness, and no significant enhancement [42].

Two technical factors that can interfere with the analysis of the vessel wall are (1) the presence of brain atrophy, in which the vessels are closer to the brain parenchyma, and this aspect can make it difficult to delimit the vessel wall [43]; (2) greater ease in detecting vessels more proximal to the circle of Willis, such as the middle cerebral artery (MCA), compared with more distal segments of the MCA, anterior and posterior cerebral artery [44].

In addition to the factors mentioned above, the use of contrast is also necessary to improve the sensitivity of vessel wall analysis, depending on the technical specifications and the patient’s clinical profile, and it can be used to assess whether treatment is effective in patients with vasculitis, as enhancement is supposed to decrease over time [45,46].

Despite the advantages of vessel wall analysis, it is important to acknowledge certain limitations. One such limitation is the use of contrast agents (iodine- and gadolinium-based), which are commonly used in conventional magnetic resonance imaging (MRI) and computed tomography (CT) but are contraindicated in patients with renal failure. A thorough clinical assessment is necessary to evaluate whether the patient has renal impairments before performing these examinations with contrast agents. In the presence of significant renal dysfunction, such as an estimated glomerular filtration rate (eGFR) below 30 mL/min per 1.73 m^2^, an alternative imaging method should be considered. Molecular imaging is a promising area of study in these situations and may become an important method in the future [44].

Angiographic imaging methods enable the identification of vascular lesions in neuropsychiatric SLE, particularly stenotic lesions. While angiography demonstrates similar sensitivity to vessel wall analysis for detecting most vascular lesions, it has lower sensitivity in evaluating small vessels, the morphology of atherosclerotic plaques, and early changes in the vascular wall. In our literature review, a significant proportion of patients who exhibited findings on vessel wall analysis did not show changes on time-of-flight (TOF) magnetic resonance angiography (MRA) [25,45]. Particularly, Ide et al. [3] found a small concordance of MRA and HRVWI, and only 4.5% of the segments with HRVWI abnormalities could be detected on TOF MRA.

**Figure 2:**
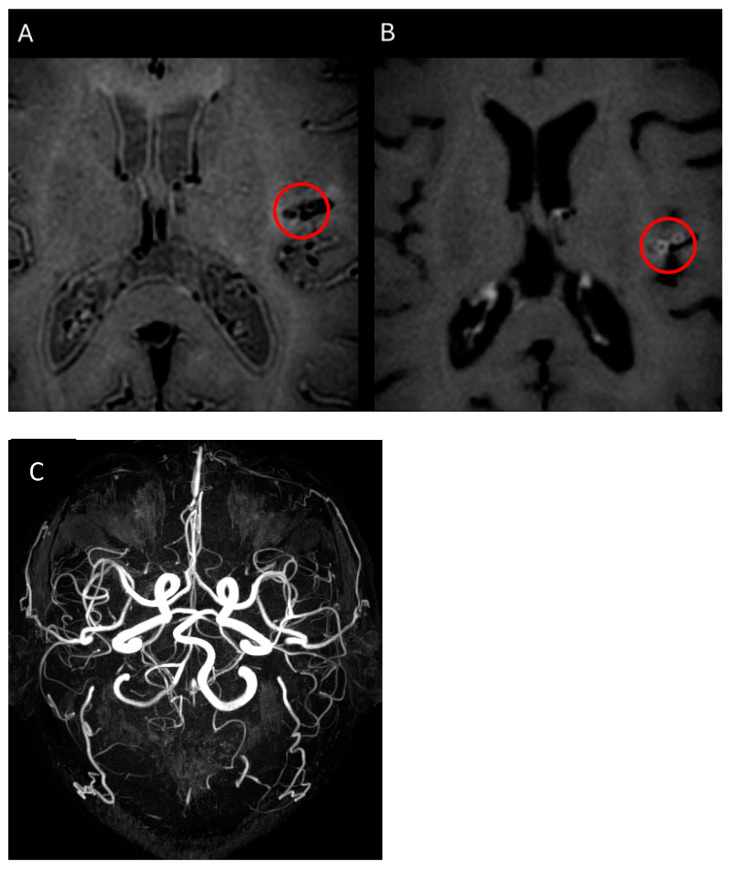
The images demonstrate the sequence of analysis of the vessel wall and its correspondence in TOF magnetic resonance angiography. Images (**A**,**B**) of the axial plane, before and after the use of contrast, show in the red circle concentric enhancement of the M2-M3 segments of the left middle brain, implying inflammatory activity (vasculitis); (**C**) an image in the 3D-TOF arterial angiographic sequence without abnormalities. Source: own archive. No copyright issue.

An additional advantage of HRVWI is its ability to differentiate between changes related to vasculitis or cerebrovascular disease. This distinction is particularly important in conditions like SLE, where multiple pathogenic mechanisms may coexist [46]. The findings from vessel wall analysis in vasculitis show wall thickening with homogeneous, concentric enhancement (and, less frequently, eccentric enhancement), generally affecting multiple segments and involving a longer path (Figure 2). In contrast, atherosclerosis also presents with wall thickening, but with eccentric enhancement that involves the circumference of the vessel wall and typically a shorter path [47]. Furthermore, Ide et al. [3] demonstrated that HRVWI abnormalities were significantly associated with brain infarctions in SLE patients, reinforcing that HRVWI is a useful tool for evaluating SLE patients.

In clinical practice, differential diagnoses for neuropsychiatric SLE should include CNS infectious diseases. These lesions affect small to large vessels and show increased enhancement and concentric parietal thickening. Eccentric parietal thickening is less common. Leptomeningeal enhancement, including cranial nerve enhancement, may also be present in infectious conditions [48,49,50]. Despite the benefits, not all patients with neuropsychiatric SLE benefit from vessel wall analysis. It is typically recommended for patients with neuropsychiatric symptoms where thromboembolic, infectious, or inflammatory causes need to be assessed. This analysis helps guide therapeutic decisions. Vessel wall analysis may also be indicated to locate and assess the activity of cerebral atherosclerotic plaques, infer activity in vasculitis (primary, SLE-related, or infectious), predict rupture risk in cerebral aneurysms, detect RCVS and differentiate it from vasculitis, and identify arterial dissection or other causes of intracranial artery narrowing [25,34,51].

Many of the characteristics highlighted in Table 2 may not be present after starting appropriate treatment. Studies point to a reduction in enhancement and an improvement in symptoms after immunosuppressive therapy [47]. The time for enhancement improvement is unclear. Studies show improvement within 1–2 weeks, but further validation is needed.

There are cases of cerebral vasculitis in SLE patients, documented on biopsy or angiography, that described successful treatment with intravenous pulse cyclophosphamide in association with corticosteroids (or not) [52,53]. Therapy for neuropsychiatric SLE depends on the associated pathophysiological mechanism. In the presence of an immune/inflammatory state, immunosuppressive treatment is generally used, with the aim of resolving/stabilizing the clinical picture, and it results in an improvement of up to 70% of the psychiatric symptoms [31,54]. Other immunosuppressive options as well as new treatments with biological therapies have also been used in SLE-related vasculitis, with variable results [29].

If the underlying pathophysiological mechanism is associated with thrombotic phenomena, antiplatelet and anticoagulant therapies can be used, including as secondary prevention. Some studies suggest that combining immunosuppressants with antiplatelet/anticoagulant agents may be beneficial [31,55]. Our review found antiplatelet use in four studies (40%).

A post-mortem brain study from NPSLE patients found that histopathological lesions represent a continuum, ranging from focal common vasculopathy to diffuse vasculitis [56].

**Table 2:** Summary of the main radiological characteristics that distinguish the main forms of intracranial vasculitis.

Vessel Wall Disease	Stenotic Pattern on Angiography, Magnetic Resonance Angiography, CT Angiography	Thickening Pattern	Localization	Enhancement
SLE central vasculitis [11,15,19,20,21,56]	Usually present	Concentric or eccentric	Different locations and longer duration	Shows enhancement before treatment
Infectious nervous vasculitis [48,49], central system	Usually present	Concentric or eccentric	Different locations and longer duration	Shows enhancement before treatment; leptomeningeal enhancement (and cranial nerve enhancement) can be observed
Intracranial atherosclerosis [57]	Can be present; vessel wall thickening or remodeling	Eccentric	Different locations and focal involvement	Could be present
Moyamoya disease [58]	Can be present	Concentric	Distal ACI and proximal ACM	Generally, no enhancement
Intracranial aneurysm [23]	Can be present	Generally without thickening	No specific location	Generally, no enhancement
Arterial dissection [17]	Present	Eccentric	Distal ICA and vertebral arteries	Usually present
RCVS [22]	Present	Concentric	Multiple locations	Generally no enhancement
After thrombectomy (iatrogenic) [59]	Can be present	Concentric or eccentric	Thrombectomy site	Usually present

## 5. Conclusions

Imaging techniques play a crucial role in neuropsychiatric SLE. Vessel wall analysis is becoming increasingly important, as it can identify early vascular changes even before alterations are visible in angiographic sequences (e.g., 3D TOF). This enables the early initiation of treatment, thereby improving the prognosis of patients with inflammatory pathologies. Further research is needed to better understand its applications and to explore how greater efficiency can be achieved through the combination of other neuroimaging diagnostic methods, particularly in the different phenotypes of neuropsychiatric SLE.

## 6. Future Perspectives

Techniques for studying the intracranial vessel wall are still being refined to make them more practical for clinical use [40,60]. Improvements in acquisition time and better suppression of cerebrospinal fluid (CSF) will enhance the visualization of small irregularities in the arterial walls and provide better characterization of changes [40,60]. Potential future applications include assessing endothelial damage after stent removal. Current studies examine the vessel wall post-procedure, but pre-procedural imaging is also needed for comparison [60]. Another application involves determining the etiology of ischemic stroke. Treatments may differ depending on the cause, especially in small-vessel disease and small subcortical infarcts, based on lesion location and characterization. It could also help identify small thrombi in a perforating artery [40]. Although vessel wall analysis is used in various scenarios, it is difficult to determine the lesion’s origin due to the lack of histological validation. In contrast, samples from extracranial arteries are obtained after endarterectomy or biopsy [40]. A study indirectly validating HRVWI enhancement patterns analyzed intracranial aneurysms and observed that enhanced patterns indicated increased cellularity and thickening of the arterial wall [61].

## 7. Limitations

This review is based mostly on case reports. We included a case of RCVS [21] because the authors considered that there was cerebral arteritis. In many cases of this review, some clinical data were not informed, but other relevant information were presented.

## Figures and Tables

**Table 1:** Characteristics and radiological findings were found in 69 patients with neuropsychiatric SLE in association with high-resolution vessel wall analysis.

Author/Year	Sex and Age (Years)	Time Between SLE Diagnosis and Symptoms (Years)	SLE Diagnostic Criteria	Neuropsychiatric SLE Symptoms	Location of Injuries	Corresponding Angiographic Findings/VWI	Highlight Pattern	Diagnosis	Treatment	Response Pattern
Jiang W, 2024 [14]	M, 23	At diagnosis	Leukopenia, C3 reduction, positive anti-DNA, anti-Sm, and ANA	Aphasia, right hemiplegia, lethargy	P	Yes	C/M	Vasculitis	MP/CYC/dual antiplatelet therapy (aspirin with clopidogrel)	PR
Silverma n A, 2023 [15]	M, 38	15	Diffuse rash, arthritis, positive antinuclear and anti-DNA antibodies	Headache with alarm signs, nausea, vomiting, and hypertension; right peripheral facial paralysis	T/BG	Yes	C/M	Vasculitis	MP/CYC	CR
Chung MS, 2021 [16]	M, 20	At diagnosis	Fever, thrombocytopenia, C3 reduction, positive antinuclear and anti-DNA antibodies	Headache, dizziness, blurred vision	BS	Yes	C/U	Aneurysm with mural thrombus and dissection	Antiplatelet / MP /hydroxychloroquine/ azathioprine	CR
Raventhi ranathan N, 2022 [17]	M, 18	N/A	ANA, anti-SSA, anti-Sm, anti-RNP	Fever, auditory and visual hallucinations, and gait changes	BG	Yes	NE	Encephalitis	MP/ plasmapheresis, Rituxima b/CYC	CR
Sarbu MI, 2020 [18]	F, 42	07	N/A	Extreme drowsiness and cognitive decline	Periventricular	No	C/M	Vasculitis	Antibiotic therapy /MP /acyclovir	NI
Nishigaic hi A, 2020 [19]	F, 62	30	Malar rash, photosensitivity, arthritis, and lupus serology	Right hemiparalysis and dysarthria	T/O/BG	Yes	C/M	Vasculitis	CYC	NI
Takeshita S, 2020 [20]	F, 47	20	Photosensitivity, arthritis, anemia, and lupus nephritis	Aphasia and right arm weakness	P/Fr/BG	Yes	C/M	Vasculitis	MP/CYC/ heparinization	CR
Chung SW, 2019 [21]	F, 35	01 month	Polyarthralgia, myalgia, facial rash, and Raynaud’s phenomenon	Headache and dizziness	Multifocal stenosis, both middle cerebral arteries, right posterior cerebral artery, and right vertebral artery	No	NE	RCVS	MP /hydroxychloroquine/calcium channel blockers	CR
Sugiyama S, 2023 [22]	F, 42	13	N/A	Fever, mental confusion, spatial neglect	P/T	No	C/M	Multifocal arterial aneurysm and stenoses	CYC/antiplatelet	CR
Satoru Ide, 2018 Pooled analysis of 60 patients [3]	Average age 39.4 (16–80) 08 M (13.3%) 52 F (86.7%)	10.5 years	N/A	N/A	Normal—37 patients Infarction—14 patients White matter hyperintensity—16 patients Arterial stenotic lesions—10 patients Lesions identified in vessel wall analysis—54 patients	Concordance of HRVWI and TOF MRI of 4.5%	554/571 segments (97%) concentric pattern 17/571 segments (3%) eccentric pattern	Segments associated with vasculitis (concentric pattern) and segments associated with atherosclerosis (eccentric pattern)	MP/immunomodulators	N/A

ANA: antinuclear antibody; BG: basal ganglia; BS: brain stem; C/M: concentric and multifocal; CR: complete resolution; C/U: concentric and unifocal; CYC: cyclophosphamide; F: female; Fr: front; HRVWI: high resolution vessel wall imaging; M: male; MP: methylprednisolone; N/A: not available; NE: no enhancement; NI: no improvement; O: occipital; P: parietal; PR: partial resolution; RCVS: reversible cerebral vasoconstriction syndrome; SLE: systemic lupus erythematosus; T: temporal; TOF: Time-of-Flight; VWI: vessel wall imaging.

## Data Availability

This study did not report any data.

## Abbreviations

The following abbreviations are used in this manuscript:

ANA	antinuclear antibody;
BG	basal ganglia;
BS	brain stem;
C/M	concentric and multifocal;
CR	complete resolution;
C/U	concentric and unifocal;
CYC	cyclophosphamide;
F	female;
Fr	front;
HRVWI	high resolution vessel wall imaging;
M	male;
MP	methylprednisolone
NA	not available;
NE	no enhancement;
NI	no improvement;
O	occipital;
P	parietal;
PR	partial resolution;
RCVS	reversible cerebral vasoconstriction syndrome;
SLE	systemic lupus erythematosus;
T	temporal;
TOF	Time-of-Flight;
VWI	vessel wall imaging.
